# The impact of internet use on the subjective age of older adults: evidence and mechanisms

**DOI:** 10.3389/fpubh.2025.1590684

**Published:** 2025-06-10

**Authors:** Zeyu Kong, Anqin Zhu

**Affiliations:** School of Government, Nanjing University, Nanjing, China

**Keywords:** subjective age, older adults, internet use, health status, self-efficacy, social capital, active aging, healthy aging

## Abstract

**Background:**

In the context of digitalization and population aging, leveraging Internet technology to reduce the subjective age of older adults helps to promote active aging and healthy aging, thereby contributing to the realization of the development of high-quality national undertakings for the aged.

**Methods:**

Data from the Chinese Longitudinal Aging Social Survey (CLASS) in 2020 were analyzed. Regression analyses and instrumental variable methods were employed to examine the main effects, heterogeneous effects, and mechanisms of Internet use on the subjective age of older adults. Latent class models and multinomial treatment effect models were used to explore the impact of Internet use patterns on the subjective age of older adults.

**Results:**

Internet use has a significant negative effect on the subjective age of older adults. This conclusion still holds after ruling out endogeneity using instrumental variables methods and conducting various robustness tests. The mechanism test shows that Internet use lowers subjective age by improving health status, self-efficacy, and social capital. Heterogeneity analysis demonstrates that the impact of Internet use on reducing subjective age is more significant among young-old individuals, those with non-agricultural hukou, and those in better economic conditions. Further analysis suggests that different Internet use patterns have varying effects on the subjective age of older adults.

**Conclusion:**

This research suggests that Internet use reduces subjective age among older adults. This effect is primarily realized through improvements in health, self-efficacy, and social capital and varies by age, hukou, economic conditions, and Internet use patterns. These findings contribute to the theoretical understanding of how Internet use can delay subjective aging and offer policy implications for enhancing the digital dividend for older adults.

## 1 Introduction

Population aging is evolving into a global challenge. As the country with the highest degree of aging, China’s older adult population and its growth rate significantly exceed the international average. From 2000 to 2022, China transitioned from an aging society to an aged society, with the number of people aged 60 and above increasing from 126 million to 280 million, and the proportion rising from 10.46 to 19.8%, with an average annual growth rate of 3.88%. The rapid aging of the population has led to a gradual decline of the demographic dividend and a continuous increase in pension security and healthcare expenditures, presenting significant challenges for economic and social development. Given this context, promoting active aging and healthy aging and enhancing the quality of life of older adults has become a critical concern for both policymakers and scholars.

Subjective age, defined as an individual’s self-perception of their age, is a crucial psychological indicator of older adults’ quality of life (1–3). Previous studies have shown that older adults perceive their subjective age as younger than their chronological age (2). A younger subjective age reflects a positive age identity and serves as an effective self-protection strategy that enables older adults to resist negative stereotypes of aging (2, 3). It has also been associated with increased economic and social participation, improved physical function, a lower incidence of chronic diseases, reduced anxiety and depressive symptoms, a lower risk of cognitive decline, decreased mortality risk, and enhanced subjective well-being (1–4). However, Chinese older adults’ subjective age is, on average, 4.8% younger than their chronological age (5), whereas this discrepancy is approximately 11% in Germany (6), 15–16% in the United States (1), and exceeds 20% in Denmark (7). Currently, China has a relatively low level of old-age security and an underdeveloped public health infrastructure, and a higher subjective age further exacerbates the disadvantaged status of older adults. Given this context, a comprehensive analysis of the mechanisms shaping subjective age is essential for developing strategies that delay subjective aging and promote well-being among older adults.

The rapid development of information and communication technologies (ICTs) has transformed older adults’ lifestyles and social interactions, offering the potential to delay subjective aging. As of 2023, there were approximately 170 million older Internet users in China, with an Internet penetration rate of 57.4% among this age group. Examining the impact of Internet use on subjective age therefore holds significant theoretical and policy relevance. Research has increasingly explored both the determinants of subjective age and the impact of Internet use among older adults. The existing literature primarily employs the bio-psycho-social model to examine the factors influencing subjective age, highlighting the significant roles of health status, psychological resources, personality traits, socioeconomic status, and social capital (8–17). However, the role of Internet use has received limited attention. Studies on Internet use among older adults have predominantly focused on its effects on health outcomes, self-efficacy, social capital, and subjective well-being (18–24). Although some research has explored the relationship between Internet/social media use and attitudes toward aging (25–27), studies directly investigating its impact on subjective age remain scarce. Consequently, further research is needed to confirm whether Internet use affects the subjective age of older adults.

The present study analyzes the effects and mechanisms of Internet use on subjective age among older adults, using data from the Chinese Longitudinal Aging Social Survey (CLASS) in 2020. This study makes several contributions. First, it introduces a new research perspective to the literature on subjective aging by integrating symbolic interactionism, social comparison theory, and social constructionism to examine the main effects of Internet use on subjective age, as well as incorporating the bio-psycho-social model to explore the underlying mechanisms. Second, it employs the instrumental variable approach to address potential endogeneity, enhancing the robustness of the findings. Third, it explores the heterogeneous effects of Internet use on subjective age and the impact of different Internet use patterns, providing empirical insights relevant to bridging the digital divide and enhancing the digital dividend for older adults.

## 2 Literature review and research hypotheses

### 2.1 Subjective age of older adults and its determinants

Subjective age refers to how old an individual perceives themselves to be, and is typically measured using felt age (3). Older adults tend to report a felt age lower than their chronological age (2). Stereotype threat theory suggests that negative age-related stereotypes can harm the behavior, health, and subjective well-being of older adults; thus, a younger subjective age can help mitigate these stereotypes and restore a sense of self-worth (2). Consequently, maintaining a younger subjective age is considered a positive self-concept that can encourage older adults to continue participating in socio-economic activities, improving their financial situation, promoting interpersonal interactions, and enhancing quality of life (1, 2). The bio-psycho-social model provides a comprehensive theoretical framework for explaining the determinants of subjective aging indicators such as subjective age (2), awareness of age-related changes (28), and subjective life expectancy (29). From an information processing perspective, this model suggests that individuals form their subjective age based on biological, psychological, and social cues about aging (2). Therefore, we categorize the determinants of subjective age into biological, psychological, and social factors.

Biological factors include various health indicators. Physical decline and cognitive impairments are key signs of biological aging; thus, worsening health is associated with an increase in subjective age among older adults. Subjective health, an individual’s perception of their health status, reflects their actual health levels. Indicators such as self-rated health and health satisfaction are associated with a lower subjective age (8, 9). Several studies have used the concept of subjective health to measure subjective aging (29, 30), which may introduce confounding biases. Consequently, related research has used objective health indicators such as activities of daily living, bodily pain, chronic diseases, BMI, depressive symptoms, and cognitive abilities to confirm that better health status is associated with a lower subjective age (8, 9). Other research has explored the relationship between biomarkers and subjective age, finding that a balanced body composition and better respiratory and muscle system function also contribute to a lower subjective age (10).

Psychological factors include psychological resources and personality traits. The stress process model suggests that aging is accompanied by negative events, such as mental decline, changes in family structure, and the loss of social relationships, which increase stress and lead to negative aging perceptions among older adults (11). Psychological resources, such as positive emotions and self-efficacy, can enhance coping abilities, mitigate the consequences of stress, and reduce subjective age. For instance, positive emotions can broaden cognitive patterns, strengthen psychological resilience, and foster a positive age identity in older adults (11). Self-efficacy—an individual’s belief in their ability to manage their life and environment and achieve their goals—encourages older adults to actively engage with society and mobilize available resources for self-regulation, reducing subjective age (12, 13). Personality traits are related to stress coping strategies and can influence vulnerability to stress and its consequences. Specifically, those with higher openness to experience and extraversion tend to adopt cognitive patterns similar to those of younger individuals, typically reporting a lower subjective age. Those with higher conscientiousness are more likely to expend extra effort when facing challenges to achieve their goals, helping to buffer against the effects of stress and delay subjective aging. Agreeableness promotes social integration, prevents feelings of loneliness, and reduces subjective age. Emotional stability can reduce perceived stress, negative aging experiences, and subjective age (14).

Social factors include socio-economic status and social capital. Continuity theory suggests that withdrawal from socio-economic activities in old age leads to role loss and a decline in status, making it difficult for subjective age to align with the middle-aged years. Socio-economic status can offset the resource losses and status decline brought about by aging and also influence the timing of role transitions. Privileged groups tend to marry, have children, and retire later, thus avoiding the “downward extension of adultlike experience” and delaying subjective aging (15). Consequently, education and income are associated with reduced subjective age, while retirement is associated with increased subjective age (5, 16). Social capital can mitigate the negative consequences of stress events (29) and compensate for the role losses associated with aging, ensuring continuity with middle-aged roles and maintaining a younger subjective age (17). Components of social capital, such as social networks, social participation, and social support, are associated with a lower subjective age (16, 17).

### 2.2 Main effects of internet use on subjective age of older adults

In the context of population aging, studies on the digital divide among older adults have explored the determinants of their access to the Internet, use pattern, and digital skills (18, 19), and have analyzed the impact of Internet use on their health status, quality of life, and self-concept (20–24). However, subjective age, as a component of self-concept, has received relatively little attention. Therefore, drawing on symbolic interactionism, social comparison theory, and social constructionism, this study explores the main effects of Internet use on older adults’ subjective age from the perspective of self-concept.

Symbolic interactionism posits that individuals construct their self-concept through interactions with others, which are based on symbols that carry shared meanings (31). Individuals convey symbols through their actions, which others then assign meaning to and provide feedback on, ultimately leading individuals to internalize others’ perceptions and form their own self-concept (3). Regarding subjective age, individuals can alter others’ perceptions by adopting behaviors typically associated with youth, thereby lowering their subjective age. The age distribution of Internet users has consistently skewed younger, with older adults often labeled “digital refugees.” This has transformed Internet use into a symbol with age-related connotations—as a typical activity for younger people, it has become a marker of youth identity (24). Thus, older adults’ Internet use can serve as a means to express age identity and establish a distinction between themselves and older adults who do not use the Internet, influencing others’ perceptions and fostering a younger subjective age (24).

Social comparison theory suggests that an individual’s self-concept is shaped by comparisons with others (32). Individuals form differentiated self-concepts by comparing themselves to others along specific dimensions. Perceiving others as superior leads to upward social comparisons, resulting in a negative self-concept. Conversely, downward social comparisons, perceiving others as inferior, foster a positive self-concept (15). Driven by self-protective motivations, older adults tend to engage in downward social comparisons, devaluing those with more visible signs of aging to elevate their own status, thereby forming a younger subjective age to counteract age-related stereotypes (33). However, the outcome of social comparisons depends on the availability of comparison targets—in the absence of inferior comparison targets, individuals may be forced to compare themselves with superior others, undermining the self-protective effect (32). The Internet increases the accessibility of information, expanding the pool of comparison targets, which may lead to more downward social comparisons and reduce subjective age. Additionally, the self-protective motivation of older adults can trigger motivated reasoning, leading them to interpret, distort, or ignore information strategically to manipulate social comparisons and improve their self-concept (32). Furthermore, the Internet’s algorithmic recommendation systems synergize with these prior motivations, creating a “beneficial online echo chamber” (34) that provides more opportunities for downward social comparisons and further lowers subjective age.

Social constructionism posits that an individual’s self-concept is shaped through public discourse construction (27). Mainstream public discourse views aging as a symbol of vulnerability, associating it with negative labels such as frailty and dependency. Individuals internalize these age-related stereotypes during their early life through literature and mass media; as they age, they associate these stereotypes with themselves, leading to self-directed ageism and increased subjective age (2). However, age-related stereotypes are not fixed. The decentralization and user-driven nature of the Internet have transformed information dissemination patterns, shifting from a top-down mass media model to a bottom-up self-media model, providing marginalized groups with opportunities for self-expression. By leveraging the Internet’s information dissemination mechanisms, older adults can negotiate, resist, and reconstruct prevailing negative and static age-related stereotypes to foster a positive age identity. For instance, older adults can use online self-presentation to counteract the negative associations between aging and physical decline or diminished attractiveness. Additionally, they can share personal experiences to highlight the diversity of the aging process and publicly expose age discrimination to advocate for the establishment of an age-friendly society, transforming the public’s negative perceptions of aging and fostering a younger subjective age (27).

Empirical research examining the impact of Internet use on older adults’ subjective age is limited. However, several studies focusing on other subjective aging indicators provide evidence for the above theories. Specifically, Cody et al. (25) found that Internet training improved older adults’ attitudes toward aging. McGrath (26) noted that middle-aged and older adults’ self-presentation on Instagram increased their visibility, helping them integrate into mainstream culture and resist ageism. Ng and Indran (27) found that older TikTok users were actively engaged in discussions about growing older, with the potential to reconstruct negative perceptions of aging. Based on this, the following hypothesis is proposed:

*H*1: Internet use can reduce the subjective age of older adults.

### 2.3 Mechanism of internet use on subjective age of older adults

The bio-psycho-social model suggests that individuals form their subjective age based on biological, psychological, and social cues. As a novel information dissemination mechanism, the Internet can generate a series of biological, psychological, and social effects, influencing the input into the subjective age formation system and, consequently, its outcomes. Based on this model and relevant empirical research, the present study proposes that Internet use lowers the subjective age of older adults by improving health status, self-efficacy, and social capital.

#### 2.3.1 Health status

Older adults’ health status can be improved through health-related Internet use (HRIU), online social interactions, and cognitive stimulation. HRIU includes activities such as online consultations, medication services, communication with fellow patients, and health information-seeking (35). Online consultations and medication services reduce the cost of offline medical visits and increase accessibility to healthcare resources, thus improving health (20); communication with fellow patients fosters social connections, helping to alleviate psychological distress caused by illness (36); and health information-seeking improves health literacy, promoting healthy behaviors and enhancing physical and mental health (35). Online social interaction involves instant messaging tools, online forums, and social media. In the context of family downsizing, online social interaction overcomes the limitations of time and space, strengthens social ties with family and friends, and alleviates loneliness and depression (21). Cognitive stimulation refers to the role of Internet use in exercising visual analysis, executive functions, and memory, thus improving cognitive functions and reducing the risk of dementia (22). As health status is a key indicator of biological aging, improving health can reduce subjective age. Thus, we propose the following hypothesis:

*H*2a: Internet use lowers the subjective age of older adults by improving health status.

#### 2.3.2 Self-efficacy

According to the concept of psychological empowerment (37), Internet use can improve older adults’ self-efficacy through several channel outcomes, including enactive attainment, vicarious experience, verbal persuasion, and emotional arousal state. First, Internet applications such as health management, online financial planning, remote education, and government services can improve older adults’ ability to live independently, helping them achieve their goals in the face of physical, psychological, and social barriers, thereby accumulating enactive mastery experiences and enhancing self-efficacy (21, 23). Second, the Internet’s information-seeking and social networking functions help older adults observe the others’ behaviors, which can promote social learning, accumulate vicarious experiences, and further enhance self-efficacy (36). Third, verbal persuasion includes advice, encouragement, and praise from others. The Internet can enhance social connections for older adults, providing them with advice and encouragement when facing difficulties, receiving praise after achieving goals, and gaining positive feedback through “likes,” “shares,” and other novel social media symbols, thereby improving self-efficacy (36). Last, the entertainment features of the Internet can alleviate fatigue, reduce anxiety, and improve emotional state, thus promoting self-efficacy (23). As a crucial psychological resource, self-efficacy can improve older adults’ coping abilities, reduce the negative consequences of stress, and lower the subjective age. Thus, we propose the following hypothesis:

*H*2b: Internet use lowers the subjective age of older adults by improving self-efficacy.

#### 2.3.3 Social capital

Internet use can improve older adults’ social capital through mechanisms such as the maintenance and expansion of social networks, the enhancement and spillover of social participation, and improvements in the quantity and quality of social support. First, the immediacy of the Internet reduces the cost of contact with others, helping to maintain existing social networks and facilitating the formation of new networks based on geographic or occupational connections, thereby expanding and constructing unfamiliar and more diverse social networks (38). Second, the Internet has introduced new communication modes, such as live streaming, video calls, and online sharing, which enhance online social interactions and improve intimate relationships (24). Additionally, because the rate of Internet addiction among older adults is low (21), online social interactions can produce offline spillover effects, further enriching social participation (24). Third, the Internet’s anonymity and de-personalized communication features can produce a disinhibition effect, thus increasing self-disclosure of painful and embarrassing experiences, enhancing trust, and leading to a greater quantity and quality of social support (36, 39). As components of social capital, social networks, social participation, and social support help mitigate the status decline and role loss caused by aging, thereby reducing subjective age. Thus, we propose the following hypothesis:

*H*2c: Internet use lowers the subjective age of older adults by improving social capital.

## 3 Methodology

### 3.1 Data

The Chinese Longitudinal Aging Social Survey (CLASS) is a nationally representative survey conducted by the National Survey Research Center at Renmin University of China. To ensure the relevance and the availability of key variables, the present study used the CLASS 2020 data for empirical analysis. CLASS 2020 covers 28 provincial-level administrative regions across China, with respondents aged 60 and above. The survey includes data on sociodemographic characteristics, health status, self-efficacy, social capital, and subjective age, making it suitable for this study. To enhance statistical power, we excluded samples with missing values in the dependent variable, independent variable, and control variables, while retaining samples with missing values in the mechanism variables. The sample size for the main effect analysis is 9,531, whereas the sample size for certain mechanism tests is smaller. Potential attrition bias arising from missing values in mechanism variables will be addressed using the inverse probability of attrition weighting (IPAW) method in subsequent analyses.

### 3.2 Variables

#### 3.2.1 Dependent variable

The dependent variable was subjective age. Following the approach used in previous literature (5), subjective age was measured as the discrepancy between felt age and chronological age. Felt age was assessed using the following question: “How old do you feel most of the time?” A larger discrepancy between felt age and chronological age indicates a higher subjective age.

#### 3.2.2 Independent variable

The independent variable was Internet use, measured as follows: never = 0, a few times per year = 1, at least once a month = 2, at least once a week = 3, and daily = 4.

#### 3.2.3 Mechanism variables

The mechanism variables included health status, self-efficacy, and social capital.

First, given that using subjective health to explain subjective age may introduce confounding biases, this study used objective health indicators, including activities of daily living (ADL), instrumental activities of daily living (IADL), chronic diseases, depressive symptoms, and cognitive ability. ADL was measured using the Katz Index, which includes six activities: feeding, dressing, bathing, toileting, continence, and transferring. The total number of activities that respondents could independently complete was used as the ADL index. IADL was measured using the Lawton Scale, which includes eight activities: using the telephone, taking medication, going out, using public transportation, shopping, handling finances, preparing food, and housekeeping. The total number of activities that respondents could independently complete was used as the IADL index. Chronic diseases were measured by the number of chronic diseases a respondent had been diagnosed with by doctors. Depressive symptoms were measured using the Center for Epidemiologic Studies Depression (CES-D) Scale, which includes nine emotions: sadness, loneliness, sorrow, disappointment, loss of appetite, sleep disturbances, worthlessness, emptiness, and loss of interest. Respondents were asked to rate these emotions on a three-point scale: never = 1, sometimes = 2, and often = 3. The total score was used as the depressive symptom index. Cognitive ability was measured using the Minimum Mental State Examination (MMSE) scale, which includes 16 items, each worth 1 point, with the total score used as the cognitive ability index.

Second, based on the approach by Ding and Wang (40), self-efficacy was measured using three items: “As I get older, I find it harder to make new friends,” “Older people are better at handling life problems,” and “I still feel like a useful person to society.” Responses range from “strongly disagree” to “strongly agree” on a five-point scale. After reverse coding the first item, the average score was used as the self-efficacy index.

Finally, the available social capital indicators in the CLASS 2020 data include social participation and social networks. Specifically, the measure of social participation was based on the frequency with which respondents engage in six activities: religious activities, training courses, watching television, singing, board games, and square dancing. The response options are coded as follows: not participating = 0, a few times a year = 1, at least once a month = 2, at least once a week = 3, and almost daily = 4. The average score was used as the social participation index. Social networks were measured using the Lubben Social Network Scale, which includes six items that focus on the number of close friends or relatives the respondent has daily contact and discusses personal matters with, and receives assistance from. Responses are scored from “none” to “9 or more” on a six-point scale, with the average score used as the social network index.

#### 3.2.4 Control variables

This study controlled for the following variables: gender (female = 0, male = 1), age (chronological age), ethnicity (Han = 0, minority = 1), religious belief (no = 0, yes = 1), hukou type (agricultural hukou = 0, non-agricultural hukou = 1), marital status (single = 0, married = 1), political status (non-CPC members = 0, CPC members = 1), education (years of education), retirement status (no = 0, yes = 1), pension (no = 0, yes = 1), economic condition (poorer = 1, average = 2, better = 3), number of houses, family size, number of children, and living arrangement (live without children = 0, live with children = 1). To account for unobservable macroeconomic confounders, province fixed effects were included in the analysis. Descriptive statistics are presented in Table 1. Respondents’ characteristics are described by means and standard deviations (SD) for numerical variables and numbers and percentages for categorical variables.

**Table 1:** Descriptive statistics.

Variables	N	Mean (SD) / %
Subjective age	9,531	−3.069 (6.460)
Internet use	9,531	1.065 (1.699)
ADL	9,531	5.692 (0.963)
IADL	9,531	7.340 (1.684)
Chronic diseases	9,531	1.639 (1.445)
Depressive symptoms	8,483	15.656 (3.291)
Cognitive ability	9,531	13.476 (3.061)
Self-efficacy	7,796	3.009 (0.710)
Social participation	9,531	0.835 (0.588)
Social networks	9,531	2.379 (0.844)
Gender		
Female	4,701	49.323%
Male	4,830	50.677%
Age	9,531	71.419 (6.566)
Ethnicity		
Han	9,014	94.576%
Minority	517	5.424%
Religious belief		
No	9,080	95.268%
Yes	451	4.732%
Hukou type		
Agricultural hukou	5,320	55.818%
Non-agricultural hukou	4,211	44.182%
Marital status		
Single	2,367	24.835%
Married	7,164	75.165%
Political status		
Non-CPC members	9,134	95.835%
CPC members	397	4.165%
Education	9,531	5.965 (4.116)
Retirement status		
No	5,373	56.374%
Yes	4,158	43.626%
Pension		
No	2,121	22.254%
Yes	7,410	77.746%
Economic condition	9,531	2.067 (0.533)
Number of houses	9,531	1.076 (0.400)
Family size	9,531	2.694 (1.308)
Number of children	9,531	2.364 (1.284)
Living arrangement		
Live without children	6,275	65.838%
Live with children	3,256	34.162%

### 3.3 Model specification

#### 3.3.1 Main effect models

This study employed the following linear regression model to examine the main effect of Internet use on the subjective age of older adults, as shown in Equation (1):


(1) $$\mathrm{Sub}\_{\mathrm{age}}_{i}=\alpha_{0}+\alpha_{1}{\text{Internet}}_{i}+\alpha_{2}X_{i}+{\text{Province}}_{i}+\varepsilon_{i},$$

where $\mathrm{Sub}\_{\mathrm{age}}_{i}\;$ denotes subjective age, ${\text{Internet}}_{i}$ represents Internet use, $X_{i}$ is a vector of control variables, ${\text{Province}}_{i}$ represents province fixed effects, and $\varepsilon_{i}$ is the error term. The coefficients $\alpha_{0}$ , $\alpha_{1}$ , and $\alpha_{2}$ are parameters to be estimated. To address potential heteroskedasticity, robust standard errors were employed in the estimation.

The above regression model may, however, suffer from endogeneity. Specifically, unobserved factors such as personality traits may influence both subjective age (14) and Internet use (41), resulting in omitted variable bias. Furthermore, as Internet use is a typical behavior of younger individuals, a lower subjective age increases the likelihood that older adults will use the Internet (30), leading to simultaneity bias. To address the potential endogeneity, we followed the approach of existing literature (42–44) and used household broadband access as an instrumental variable, constructing the following two-stage least squares (2SLS) model. The first-stage is specified as Equation (2) and the second-stage is specified as Equation (3):


(2) $${\text{Internet}}_{i}=\beta_{0}+\beta_{1}{\text{Broadband}}_{i}+\beta_{2}X_{i}+{\text{Province}}_{i}+\zeta_{i}$$

(3) $$\begin{array}{c} \mathrm{Sub}\_{\mathrm{age}}_{i}=\gamma_{0}+\gamma_{1}\hat{{\text{Internet}}_{i}}+\gamma_{2}X_{i}+{\text{Province}}_{i}+\eta_{i}, \end{array}$$

where ${\text{Broadband}}_{i}$ is the instrumental variable, and $\hat{{\text{Internet}}_{i}}$ is the fitted value of Internet use obtained from the first-stage regression (Equation 2). Since household broadband access is a prerequisite for Internet use, it satisfies the strong first-stage condition. Additionally, broadband access is assumed to affect subjective age only indirectly through its influence on Internet use among older adults, satisfying the exclusion restriction. Previous studies have also employed similar instrumental variables to address the endogeneity of Internet use. For example, Zhang and Li (42), using data from the 2017 Chinese General Social Survey (CGSS), employed household Internet connectivity as an instrumental variable to estimate the impact of Internet use on older adults’ social networks. Ma et al. (43), based on data from the 2018 and 2020 China Health and Retirement Longitudinal Study (CHARLS), used broadband access as an instrumental variable to estimate the effect of Internet use on middle-aged and older adults’ self-rated health. Zhang et al. (44), using survey data from four Chinese cities in 2019, employed Internet accessibility as an instrumental variable to estimate the impact of Internet use on the mental health of older adults. Taken together, these examples demonstrate the plausibility of using broadband access as an instrumental variable for Internet use in the present study. In the following empirical analysis, we further tested the validity of the instrumental variable through weak instrument tests and randomization inference (45). We also assessed the robustness of the 2SLS estimates using the union of confidence intervals (UCI) and the local-to-zero (LTZ) methods. Therefore, $\gamma_{1}$ provides an unbiased estimate of the causal effect of Internet use on subjective age of older adults.

#### 3.3.2 Mechanism analysis models

To examine the mechanisms by which Internet use impacts the subjective age of older adults, we followed the approach of existing studies (46, 47) and constructed the following stepwise regression models. The model for the mechanism variable is presented in Equation (4), while the model for the dependent variable incorporating the mechanism variable is specified in Equation (5):


(4) $$\begin{array}{c} M_{i}=a_{0}+a_{1}\hat{{\text{Internet}}_{i}}+a_{2}X_{i}+{\text{Province}}_{i}+\mu_{i} \end{array}$$

(5) $$\begin{array}{c} \mathrm{Sub}\_{\mathrm{age}}_{i}=b_{0}+b_{1}\hat{{\text{Internet}}_{i}}+b_{2}M_{i}+b_{3}X_{i}+{\text{Province}}_{i}+\xi_{i}, \end{array}$$

where $M_{i}$ represents the mechanism variables, including health status, self-efficacy, and social capital; $\hat{{\text{Internet}}_{i}}$ is the fitted value of Internet use from Equation 2; coefficient $a_{1}$ captures the effect of Internet use on the mechanism variables; $b_{1}$ represents the direct effect of Internet use on subjective age; and $b_{2}$ measures the effect of the mechanism variables on subjective age. If both $a_{1}$ and $b_{2}$ are statistically significant and $b_{1}$ is smaller than the Internet use coefficient in the main effect model, this provides evidence that Internet use influences subjective age through the specified mechanism variable.

It is worth noting that due to missing values for the depressive symptoms and self-efficacy measures, the sample size for their corresponding mechanism analysis models is smaller than that of the main effect model, which may introduce attrition bias. To address this issue, we adopted the IPAW method, following Falisse et al.’s (48) approach, to adjust for the imbalances between the full and attrition samples. Specifically, we estimated a probit model to predict the denominator of the weight using the independent variables, control variables, and province dummies, and then excluded covariates that are significantly correlated with the probability of attrition to predict the numerator of the weight. This ensures the representativeness of the mechanism analysis results and enhances their comparability with the main effect analysis.

#### 3.3.3 Further analysis models

Given the heterogeneity in Internet use patterns among older adults, it is necessary to explore how different patterns affect subjective age. To this end, we first constructed a latent class model to classify Internet use patterns, using 11 types of online activities as observed indicators: voice and video chatting, text chatting, online shopping, reading news, browsing information, playing music and video, playing games, transportation and travel, health management, financial management, and learning and training. The optimal number of latent classes was determined using multiple criteria, including the Akaike information criterion (AIC), Bayesian information criterion (BIC), sample-size adjusted BIC (SSABIC), entropy, and the *p*-values from the Lo–Mendell–Rubin likelihood ratio test (LMR) and the bootstrap likelihood ratio test (BLRT). This classification yields the Internet use pattern variable.

We then estimated the effect of Internet use patterns on the subjective age of older adults by employing a multinomial treatment effect model (MTEM), using non-Internet users as the reference group and broadband access as an instrumental variable. The MTEM is estimated via a two-stage regression procedure. In the first stage, we estimated a mixed multinomial logit model to assess the determinants of Internet use patterns, as shown in Equation (6):


(6) $$\begin{array}{c} \text{Patter}n_{\mathrm{ij}}^{*}=c_{0}+c_{1}Z_{i}+c_{2}l_{\mathrm{ij}}+c_{3}{\text{Broadband}}_{i}+\varphi_{\mathrm{ij}} \end{array}$$

where subscript j refers to a specific Internet use pattern, $\text{Patter}n_{\mathrm{ij}}^{*}$ denotes the latent Internet use patterns, $Z_{i}$ includes control variables and province dummies, $l_{\mathrm{ij}}$ represents latent variables influencing Internet use patterns, and ${\text{Broadband}}_{i}$ is the instrumental variable.

In the second stage, we estimated the impact of Internet use patterns on subjective age using linear regression model, as shown in Equation (7):


(7) $$\begin{array}{c} E(\mathrm{Sub}\_{\mathrm{age}}_{i}|{\text{Pattern}}_{i},Z_{i},l_{i})=d_{0}+d_{1}Z_{i}+\sum_{j}d_{j}\text{Patter}n_{\mathrm{ij}}+\sum_{j}d_{j}l_{\mathrm{ij}}. \end{array}$$

Here, the latent variable $l_{\mathrm{ij}}$ , assumed to follow a standard normal distribution, captures unobserved heterogeneity that may lead to endogeneity. The two-stage model was jointly estimated using maximum simulated likelihood, yielding consistent estimates of the effects of Internet use patterns on subjective age of older adults.

## 4 Results

### 4.1 Baseline regression analysis

Table 2 presents the baseline regression analysis. Column 1 includes the core explanatory variable, while columns 2–4 successively incorporate individual, family, and provincial level control variables. The full model in column 4 shows that Internet use significantly negatively affects subjective age, supporting hypothesis H1. Regarding the control variables, age significantly negatively affects subjective age. Due to the internalization of aging stereotypes as chronological age increases, felt age will become younger than chronological age, driven by self-protection motivations, leading to a decrease in subjective age (5). Religious belief significantly negatively affects subjective age. Religion helps older adults form positive life attitudes, encourages them to adopt a regular lifestyle, and promotes participation in religious activities, improving physical and mental health, enhancing social participation, and reducing subjective age. Hukou type significantly negatively affects subjective age. The segmentation of Hukou exacerbates the economic vulnerability of rural older adults and increases their subjective age. Marital status significantly negatively affects subjective age. Marriage relationships compensate for the loss of social relationships caused by aging, increase social support, enrich psychological resources, and reduce subjective age. Political status significantly negatively affects subjective age. CPC membership, as a form of political capital, can increase income, expand social networks, and lower subjective age. Family size significantly positively affects subjective age. A larger family size may lead older adults to focus on household activities, crowd out social participation, and potentially provoke family conflicts, increasing stress levels, thus raising subjective age. The effects of the remaining control variables are non-significant.

**Table 2:** Baseline regression analysis.

Variables	(1)	(2)	(3)	(4)
Internet use	−0.094^***^ (0.036)	−0.339^***^ (0.042)	−0.365^***^ (0.042)	−0.411^***^ (0.042)
Gender		0.021 (0.133)	0.020 (0.133)	0.089 (0.131)
Age		−0.237^***^ (0.013)	−0.230^***^ (0.013)	−0.249^***^ (0.013)
Ethnicity		1.165^***^ (0.231)	1.099^***^ (0.232)	−0.008 (0.245)
Religious belief		−2.266^***^ (0.399)	−2.277^***^ (0.395)	−0.634^*^ (0.373)
Hukou type		−0.273 (0.200)	−0.509^**^ (0.204)	−0.341^*^ (0.201)
Marital status		−0.545^***^ (0.158)	−0.727^***^ (0.192)	−0.416^**^ (0.189)
Political status		−1.219^***^ (0.366)	−1.345^***^ (0.367)	−0.989^***^ (0.367)
Education		−0.058^***^ (0.019)	−0.063^***^ (0.019)	−0.028 (0.019)
Retirement status		0.116 (0.211)	0.068 (0.211)	−0.012 (0.219)
Pension		0.457^**^ (0.181)	0.435^**^ (0.181)	−0.265 (0.199)
Economic condition		−0.109 (0.130)	−0.121 (0.129)	0.068 (0.125)
Number of houses			0.841^***^ (0.181)	0.284 (0.183)
Family size			0.374^***^ (0.099)	0.171^*^ (0.102)
Number of children			−0.169^***^ (0.062)	0.001 (0.063)
Living arrangement			−0.231 (0.294)	0.166 (0.296)
Constant	−2.969^***^ (0.082)	15.029^***^ (0.993)	13.404^***^ (1.008)	13.624^***^ (1.032)
Province fixed effects	No	No	No	Yes
N	9,531	9,531	9,531	9,531

*p < 0.1, **p < 0.05, ***p < 0.01; Robust standard errors in parentheses.

### 4.2 Endogeneity test

To address the endogeneity issue in the baseline regression model, this study followed the approach of existing literature (42–44), employing household broadband access as an instrumental variable and constructing a two-stage least squares (2SLS) model to estimate the effect of Internet use on older adults’ subjective age. The first-stage regression results presented in Table 3 show that the instrumental variable significantly positively affects Internet use. The Kleibergen–Paap Wald rk F-statistic is 2678.121, which is greater than the critical value of 16.38 at the 10% significance level. The Anderson–Rubin Wald test results are significant, rejecting the null hypothesis that the sum of the regression coefficients of the endogenous variables equals zero, indicating no weak instrument problem. The second-stage regression results presented in Table 3 show that Internet use significantly negatively affects subjective age, confirming that the baseline findings still hold after ruling out endogeneity. The Durbin–Wu–Hausman (DWH) test results are also significant, indicating that Internet use is an endogenous variable and that the baseline regression analysis results suffer from downward bias, while the 2SLS results provide unbiased estimates. The instrumental variable regression results are presented in the following analysis.

**Table 3:** Endogeneity test.

Variables	Reduced-form	First-stage	Second-stage
Internet use			−0.204^**^ (0.093)
Instrumental variable	−0.329^**^ (0.150)	1.611^***^ (0.031)	
Control variables	Yes		
Province fixed effects	Yes		
N	9,531		
Kleibergen-Paap rk Wald F statistic	2678.121		
Anderson-Rubin Wald test	4.820^**^		
DWH test	5.963^**^		
UCI test	[−0.930, −0.022]		
LTZ test	−0.476^***^ (0.160)		

*p < 0.1, **p < 0.05, ***p < 0.01; Robust standard errors in parentheses.

**Figure 1:**
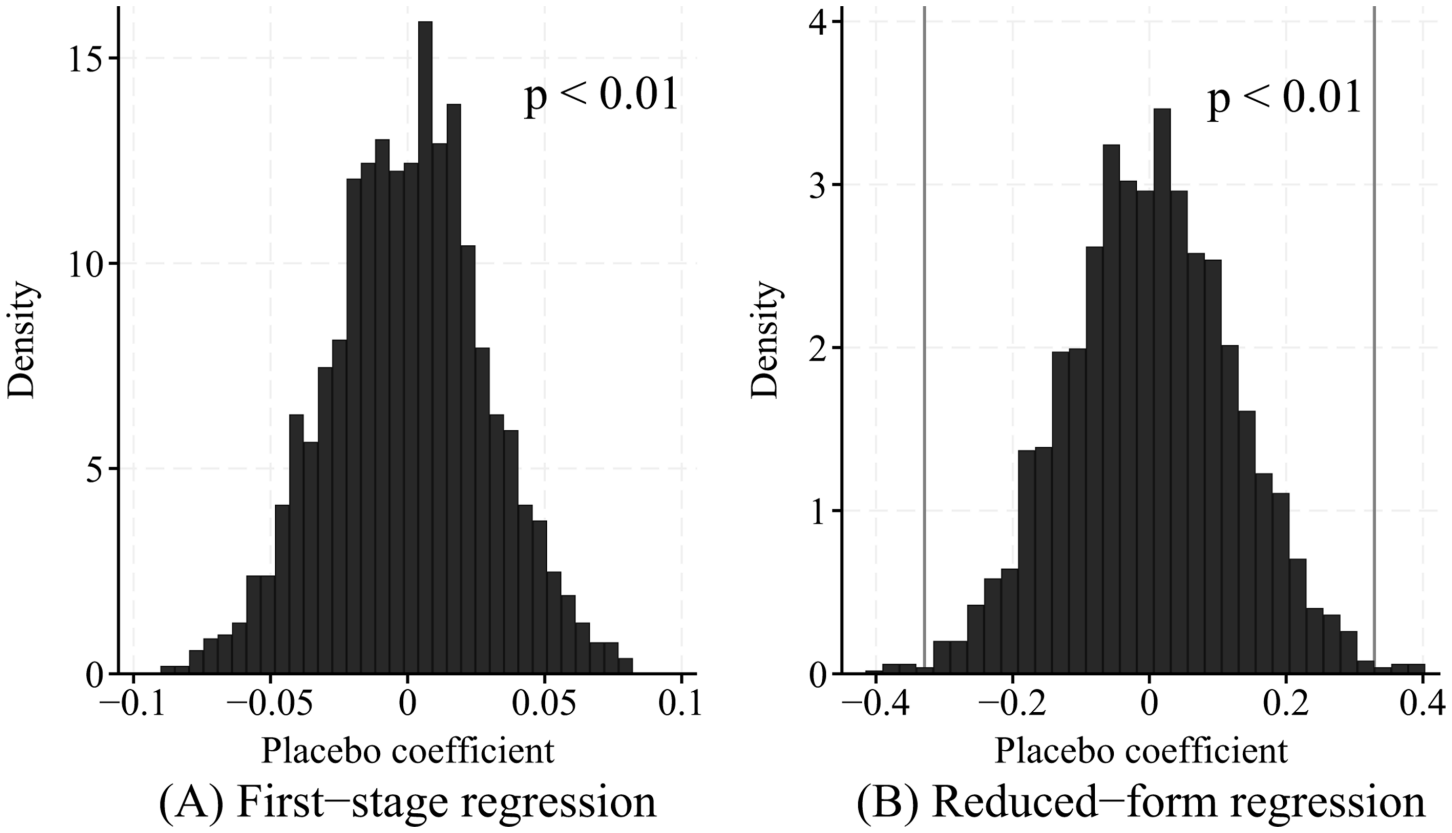
Randomization inference. The panels plot histograms of the placebo coefficients of instrumental variable. Panel **(A)** examine the first-stage placebo effect of instrumental variable on Internet use. Panel **(B)** examine the reduced-form placebo effect of instrumental variable on subjective age. The *p*-value reflects the share of placebo coefficients with absolute values exceeding that of the actual coefficient, i.e., the portions beyond the gray vertical lines. Given that the first-stage placebo coefficient is substantially smaller than the actual coefficient, the gray vertical line is omitted in Panel **(A)**.

Since the number of instrumental variables equals the number of endogenous variables, an overidentification test cannot be conducted. Following the approach of Chen et al. (45), this study employed a randomization inference method to assess the exclusion restriction of the instrumental variable. Specifically, placebo values for the instrumental variable were randomly generated, and their effects on Internet use (i.e., the first-stage regression) and subjective age (i.e., the reduced-form regression) were estimated. This procedure was repeated 2,000 times, yielding 2,000 placebo coefficients for each regression. Figure 1 presents histograms of the placebo estimates from the first-stage and reduced-form regressions. The results indicate that the placebo instrument has much less power to predict both Internet use and subjective age than the actual instrument, with the majority of placebo coefficients being close to zero. Evaluated by the *p*-value, fewer than 1% of the placebo coefficients have an absolute value larger than that of the actual coefficient, in both the first-stage and reduced-form regressions. These findings suggest that the instrumental variable is unlikely to affect subjective age through channels other than Internet use.

To further assess the robustness of the 2SLS estimates, this study used the UCI approach and the LTZ to test the sensitivity of the 2SLS results when the instruments are plausibly exogenous. The UCI test shows that the 95% confidence interval for the Internet use coefficient is [−0.930, −0.022], which does not include zero. The LTZ test reveals that the Internet use coefficient is significantly negative. Therefore, the estimated results remain robust even when relaxing the exclusion restriction of the instrumental variable.

### 4.3 Robustness checks

#### 4.3.1 Substitution of dependent variable

In the previous analysis, the dependent variable was measured as the discrepancy between felt age and chronological age. Here, following relevant literature (1, 5, 16), the dependent variable was replaced with felt age (SA_1_), the ratio of the discrepancy between felt age and chronological age to chronological age (SA_2_), and whether felt age is greater than or equal to chronological age (SA_3_). The 2SLS and IV-Probit models were used for regression. Panel A of Table 4 shows that Internet use significantly negatively affects all different subjective age indicators, validating the robustness of the previous findings.

**Table 4:** Robustness checks.

Methods	Results	Control variables	Province fixed effects	N
Panel A. Substitution of Dependent Variable				
SA_1_, 2SLS	−0.204^**^ (0.093)	Yes	Yes	9,531
SA_2_, 2SLS	−0.003^**^ (0.001)	Yes	Yes	9,531
SA_3_, IV-Probit	−0.125^***^ (0.020)	Yes	Yes	9,531
Panel B. Winsorization of Dependent Variable				
Winsorized at 0.5% levels	−0.181^**^ (0.089)	Yes	Yes	9,531
Winsorized at 1% levels	−0.174^**^ (0.087)	Yes	Yes	9,531
Winsorized at 5% levels	−0.151^**^ (0.072)	Yes	Yes	9,531
Panel C. Correction for Self-Selection Bias				
PSM, Nearest-neighbor matching	−1.115^***^ (0.288)	Yes	Yes	9,479
PSM, Radius matching	−1.476^***^ (0.251)	Yes	Yes	9,479
PSM, Kernel matching	−1.473^***^ (0.240)	Yes	Yes	9,479
IPW	−1.477^***^ (0.188)	Yes	Yes	9,531
ESR	−0.978^***^ (0.037)	Yes	Yes	9,531
Panel D. Boundary Analysis				
Test 1	3.985	Yes	Yes	9,531
Test 2	−0.562^***^ (0.054)	Yes	Yes	9,531

*p < 0.1, **p < 0.05, ***p < 0.01; Robust standard errors in parentheses.

#### 4.3.2 Winsorization of dependent variable

To avoid the impact of extreme values of the dependent variable on the regression results, subjective age was winsorized at the 0.5, 1, and 5% levels from both tails, followed by a 2SLS regression. Panel B of Table 4 shows that Internet use still significantly negatively affects subjective age, confirming the robustness of the previous findings.

#### 4.3.3 Correction for self-selection bias

The previous analysis constructed the independent variable based on older adults’ Internet use frequency. However, there may be systematic differences between Internet users and non-users that could also be related to subjective age, leading to self-selection bias. To address this, Internet use was converted into a binary variable (non-users = 0, users = 1), and methods including propensity score matching (PSM), inverse probability weighting (IPW), and endogenous switching regression (ESR) were used to estimate the average treatment effect on the treated (ATT). Specifically, the PSM method was used to estimate the propensity scores of Internet use based on observed covariates and employed nearest-neighbor matching, radius matching, and kernel matching techniques to match Internet users with non-users who had similar propensity scores, thereby estimating the ATT. The IPW method was used to calculate weights for Internet users and non-users based on their propensity scores to balance the characteristics between them and estimate the ATT through weighted regression analysis. For the ESR method, household broadband access was used as an instrumental variable, and maximum likelihood estimation was employed to predict the counterfactual values of subjective age for both groups, thus enabling the estimation of ATT. Among these, PSM and IPW can correct for self-selection bias caused by observable factors; however, the former may exclude non-overlapping samples, while the latter does not lead to sample loss. ESR can overcome self-selection bias caused by both observable and unobservable factors. Panel C of Table 4 shows that the ATT obtained by different methods is significantly negative. After correcting for self-selection bias, the above conclusion still holds.

#### 4.3.4 Boundary analysis

The previous analysis may be affected by unobservable factors that influence Internet use and subjective age simultaneously. Although the instrumental variable method can rule out this bias, we further assessed the impact on the estimation results using the boundary analysis approach. Boundary analysis is used to assess the severity of omitted variable bias by examining the stability of regression coefficients. This method requires two key pieces of information: (1) R_max_, which represents the maximum R^2^ obtained from a hypothetical regression including both observed and unobserved variables, and (2) *δ*, which represents the explanatory power of unobserved variables relative to observed factors. Boundary analysis can be conducted in two ways: by calculating the value of *δ* required to reduce the treatment effect to zero given a specified R_max_ (if |δ| > 1, the test is considered passed); and by calculating the treatment effect under specified values of R_max_ and δ (if the effect remains significantly negative, the test is considered passed). Following the recommendations of Oster (49), this study set R_max_ to 1.3 times the R^2^ from the baseline regression and assumed δ = 1, implying that the impact of unobservable variables equals that of the observed factors. Panel D of Table 4 shows that the δ from the first test is −3.985, with |δ| > 1, and the treatment effect obtained from the second test is significantly negative. Therefore, even in the presence of unobservable omitted variables, the baseline regression results remain valid.

### 4.4 Mechanism analysis

The above analyses suggest that Internet use can significantly reduce the subjective age of older adults. To shed light on these results, we attempted to test the potential mechanisms, guided by our theoretical framework. Specifically, we explored the set of possible channel outcomes: health status, self-efficacy, and social capital.

#### 4.4.1 Health status

**Figure 2:**
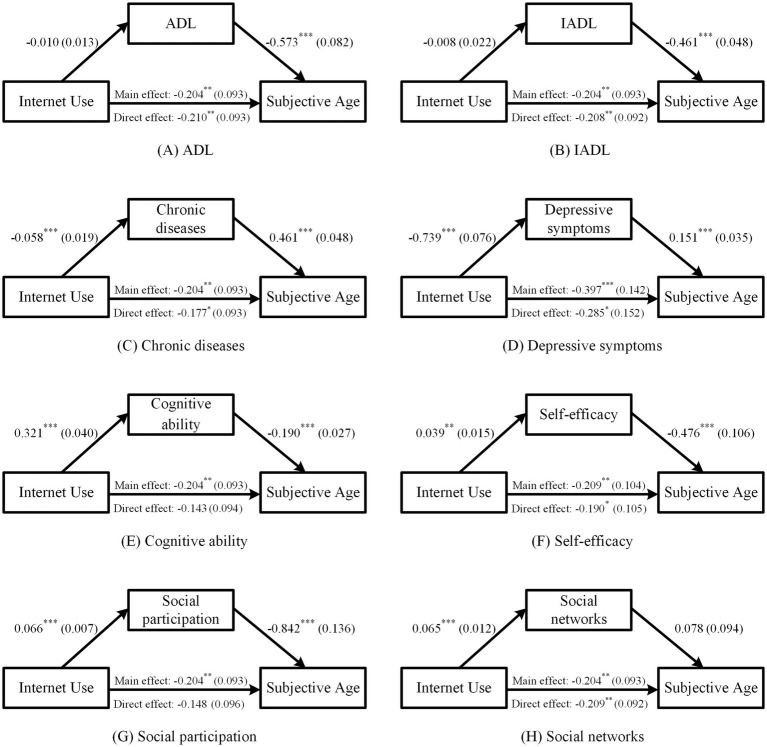
Mechanism analysis. Panels **(A)** to **(H)** present the mechanism analysis results for the following variables, respectively: ADL, IADL, chronic diseases, depressive symptoms, cognitive ability, self-efficacy, social participation, and social networks. The panels plot the results of the mechanism analysis for different mechanism variables, including the effect of Internet use on the mechanism variables, the effect of the mechanism variables on subjective age, and both the main and direct effects of Internet use on subjective age. Since the mechanism analysis models for depressive symptoms and self-efficacy used the IPAW method to correct for attrition bias, the main effect coefficients in Panels **(D)** and **(F)** differ from those in Panels **(A)**–**(C)**, **(E)**, **(G)**, and **(H)**.

Figures 2A–E indicate that Internet use significantly negatively affects chronic diseases and depressive symptoms, significantly positively affects cognitive ability, but has no significant effect on ADL and IADL. A possible explanation for this is that Internet use primarily improves older adults’ health status by facilitating access to health information and medical services, enhancing social interaction, and providing cognitive stimulation. However, impairments in ADL and IADL are often the result of biological aging and accidental injuries, which are typically irreversible and difficult to recover from in the short term. Given that we rely on cross-sectional data with a limited observation window, Internet use may not have an immediate mitigating impact on functional impairments among older adults, resulting in non-significant effects on ADL and IADL. When chronic diseases, depressive symptoms, and cognitive ability are included in the model, the effect of Internet use on subjective age weakens. However, the effect of Internet use on subjective age remains unchanged when ADL and IADL are included. Therefore, Internet use can reduce subjective age by lowering the incidence of chronic diseases, alleviating depressive symptoms, and enhancing cognitive ability, supporting hypothesis H2a.

#### 4.4.2 Self-efficacy

Figure 2F demonstrates that Internet use significantly positively affects self-efficacy. After including self-efficacy in the model, the effect of Internet use on subjective age weakens. Therefore, Internet use can reduce subjective age by enhancing self-efficacy, supporting hypothesis H2b.

#### 4.4.3 Social capital

Figures 2G,H show that Internet use significantly positively affects social participation and social networks. When social participation is included in the model, the effect of Internet use on subjective age weakens. However, when social networks are included, the effect of Internet use on subjective age remains unchanged, while the effect of social networks on subjective age is non-significant. This may be because the measurement of social networks was based on the Lubben Social Network Scale, which evaluates the friends and relatives older adults engage in daily interactions and discuss personal matters with, and receive assistance from. Social support obtained through social networks may enhance resource endowment, compensate for role loss, and reduce subjective age. However, it could also increase dependence on others, exacerbate feelings of uselessness, and elevate subjective age. The overall impact of social networks on subjective age is therefore non-significant. To summarize, Internet use can reduce subjective age by promoting social participation, supporting hypothesis H2c.

### 4.5 Heterogeneity analysis

Older adults represent a highly heterogeneous group, and the impact of Internet use on their subjective age may vary depending on individual characteristics. Accordingly, we employed the instrumental variable methods to further explore heterogeneous treatment effects across older adults, specifically across age, hukou type, and economic conditions.

#### 4.5.1 Age heterogeneous effects

Panel A of Table 5 shows that Internet use significantly negatively affects the subjective age of the young-old group, but has no significant effect on the old-old group. This may be because the young-old group has better health status and fewer barriers to Internet use, making them more likely to benefit from it. In contrast, the health decline of the old-old group reduces their ability to use the Internet, making it difficult to utilize its benefits. Furthermore, the old-old group is more likely to fall victim to online fraud, increasing financial pressure and preventing Internet use from reducing subjective age (36). Additionally, compared with the old-old group, the young-old group perceives the Internet as being more useful (20), which may create an age-conditioned positive selection, making Internet use more effective in reducing their subjective age.

**Table 5:** Heterogeneity analysis.

Subgroups	Internet use	Control variables	Province fixed effects	N
Panel A. Age Heterogeneous Effects				
Young-old group, ages 60 to 69 years	−0.360^***^ (0.108)	Yes	Yes	4,327
Old-old, ≥70 years	0.055 (0.172)	Yes	Yes	5,204
Panel B. Hukou Type Heterogeneous Effects				
Agricultural hukou	−0.103 (0.128)	Yes	Yes	5,320
Non-agricultural hukou	−0.255^*^ (0.137)	Yes	Yes	4,211
Panel C. Economic Condition Heterogeneous Effects				
Poorer	−0.538 (0.336)	Yes	Yes	1,056
Average	−0.064 (0.106)	Yes	Yes	6,778
Better	−0.494^**^ (0.221)	Yes	Yes	1,697

*p < 0.1, **p < 0.05, ***p < 0.01; Robust standard errors in parentheses.

#### 4.5.2 Hukou type heterogeneous effects

Panel B of Table 5 shows that Internet use significantly negatively affects the subjective age of older adults with non-agricultural hukou but has no significant effect on those with agricultural hukou. This may be due to the relatively underdeveloped digital infrastructure in rural areas, with lower household broadband coverage and fewer fixed computers. Rural older adults typically access the Internet via mobile networks and smartphones, which are slower than fiber-optic and cable networks. Additionally, smartphones have lower performance compared to fixed computers (50), which limits the effective use of the Internet by rural older adults, preventing Internet-induced reductions in their subjective age. In contrast, urban older adults have earlier access to the Internet, more experience, and better digital skills, allowing them to utilize the Internet effectively. Thus, Internet use is more beneficial in reducing the subjective age of older adults with non-agricultural hukou.

#### 4.5.3 Economic condition heterogeneous effects

Panel C of Table 5 shows that Internet use significantly negatively affects the subjective age of older adults with better economic conditions, but has no significant effect on those living in poorer or average economic conditions. These results can be explained by three factors: internet access devices, network infrastructure, and online services. First, older adults with better economic conditions can afford larger and better-equipped devices, which enhances the effectiveness of their use. Second, wealthier older adults typically reside in areas with better network infrastructure and can afford the costs of home broadband and data plans, allowing them to access more extensive online content and improve the quality of their Internet use. Third, the increasing trend toward paid Internet content creates disparities in the quality of online services accessed by different economic groups. Those with better economic conditions can afford high-quality paid content, while those living in poverty or average economic conditions tend to access homogeneous free content, exacerbating inequalities in the effectiveness of Internet use. Consequently, Internet use is more effective in reducing the subjective age of older adults living in better economic conditions.

### 4.6 Further analysis

According to research on the digital divide, there are three types of gaps in Internet use across different groups. The first-level digital divide, also known as the access gap, refers to disparities among different groups in terms of access to the Internet. The second-level digital divide, referred to as the usage gap, pertains to differences in Internet use motivation, content engagement, and preferences among different groups. The third-level digital divide, termed the utility gap, represents inequalities in the consequences of Internet use across different groups (51). The previous sections explored the main and heterogeneous effects of Internet use on the subjective age of older adults from the perspectives of the access and utility gaps. Here, we present an analysis of the effects of different Internet use patterns on subjective age, based on the concept of the usage gap, thereby clarifying the complex impact of the digital divide.

Following the approach of Pantelaki et al. (52), we constructed a latent class model to classify Internet use patterns using 11 types of online activities as observable variables. Table 6 shows that the AIC and SSABIC decrease, while the BIC first decreases and then increases, suggesting that the 5- and 6-class models fit the data well. The entropy for both the 5- and 6-class models is around 0.7, indicating that the classification accuracy is acceptable. The BLRT test for the 6-class model is significant at the 0.1% level, but the LMR test is only significant at the 1% level. In the 5-class model, all class proportions are above 10%, whereas in the 6-class model, the probability of class 1 is below 5%, indicating that the classification is too detailed and lacks sufficient discrimination (53). Therefore, the 5-class model was selected as the optimal model.

**Table 6:** Latent class models.

Latent class	AIC	BIC	SSABIC	Entropy	LMR	BLRT	Class proportions
2	28579.169	28718.203	28645.122	0.773	<0.001	<0.001	26.491%/73.509%
3	28097.412	28308.985	28197.775	0.697	<0.001	<0.001	38.775%/49.519%/11.706%
4	27892.774	28176.887	28027.548	0.728	<0.001	<0.001	39.609%/21.745%/ 25.529%/13.117%
5	27801.055	28157.707	27970.239	0.693	<0.001	<0.001	12.989%/24.792%/ 18.249%/29.570%/14.400%
6	27734.399	28163.591	27937.994	0.731	0.006	<0.001	4.362%/36.979%/19.917%/ 10.295%/8.499%/19.949%

Table 7 displays the response probabilities of observable variables for the 5-class model. In class 1, the response probabilities of all activities are relatively high, and it is labeled “comprehensive users.” In class 2, the response probabilities of online chatting, information browsing, and leisure entertainment are higher than those of other activities, and it is labeled “social-information-entertainment users.” In class 3, the response probabilities of online chatting and news watching are higher than those of other activities, and it is labeled “social-news users.” In class 4, the response probabilities of online chatting, news watching, and media playback are higher than those of other activities, and it is labeled “social-news-audiovisual users.” In class 5, the response probability of online chatting is significantly higher than that of other activities, and it is labeled “social users.”

**Table 7:** Response probabilities of observable variables in 5-class model.

Variables	Class 1	Class 2	Class 3	Class 4	Class 5
	Comprehensive users	Social-information-entertainment users	Social-news users	Social-news-audiovisual users	Social users
Voice and video chatting	0.981	1	0.981	0.749	1
Text chatting	0.979	0.886	0.984	0.122	0.243
Online shopping	0.872	0.266	0.253	0.076	0
Reading news	0.897	0.753	0.760	0.587	0.093
Browsing information	0.904	0.694	0.349	0.254	0
Playing music and video	0.891	1	0.215	0.520	0.248
Playing games	0.359	0.472	0.126	0.102	0.006
Transportation and travel	0.898	0.089	0.096	0.056	0
Health management	0.443	0.022	0.026	0.012	0
Financial management	0.264	0.004	0.058	0.010	0
Learning and training	0.077	0.004	0.001	0.006	0.001

Then, we took non-Internet users as the reference group and household broadband access as the instrumental variable to construct a multinomial treatment effect model (MTEM) to examine the impact of Internet use patterns on subjective age. Table 8 shows that the comprehensive, social-news, and social-news-audiovisual users significantly negatively affect subjective age, while the effects of the social-information-entertainment and social users are not significant. The possible reasons for these results are as follows: First, compared to other users, comprehensive users cover the broadest range of online activities, with higher response probabilities for instrumental uses such as transportation, health management, and financial management. This helps enrich their digital experiences, provides convenience in daily life, and enhances health and economic capital, thereby reducing subjective age. Second, social-news users include social interactions and information acquisition activities, allowing them to access high-quality health information, strengthen social connections with family and friends, and accumulate vicarious experiences (36). This improves health and self-efficacy, leading to a reduction in subjective age. However, the response probability of instrumental activities in this class is lower, making its effect on subjective age weaker than that of the comprehensive users. Third, social-news-audiovisual users also involve social interactions and information acquisition, but their entertainment activities are more time-consuming, making it difficult to achieve capital enhancement, thereby offsetting the effects of social and informational activities and weakening the impact on subjective age compared to social-news users. Fourth, social-information-entertainment users have the highest response probability for entertainment activities, which can lead to unhealthy behaviors such as prolonged screen time or sedentary lifestyles, triggering Internet dependency and harming users’ physical and mental health, making a reduction in subjective age less likely. Finally, social users involve the fewest digital activities, almost entirely excluding information and instrumental uses, preventing them from fully benefiting from the Internet and thus failing to reduce subjective age.

**Table 8:** Multinomial treatment effect model.

Variables	(1)
Comprehensive users	−3.007^***^ (0.442)
Social-information-entertainment users	−0.231 (0.720)
Social-news users	−2.051^**^ (0.964)
Social-news-audiovisual users	−1.114^***^ (0.426)
Social users	−0.719 (0.495)
Control variables	Yes
Province fixed effects	Yes
N	9,531

*p < 0.1, **p < 0.05, ***p < 0.01; Robust standard errors in parentheses.

## 5 Conclusion and policy implications

Using data from the China Longitudinal Aging Social Survey in 2020, this study empirically examined the effects and mechanisms of Internet use on the subjective age of older adults. The findings are as follows: First, Internet use significantly reduces the subjective age of older adults. Second, Internet use lowers subjective age by improving health status, self-efficacy, and social capital. Third, the impact of Internet use on reducing subjective age is more significant among individuals aged 60–69 (i.e., the young-old individuals), those with non-agricultural hukou, and those with better economic conditions. Fourth, the impact of different Internet use patterns on subjective age varies. Specifically, comprehensive, social-news, and social-news-audiovisual significantly reduce their subjective age, while social-information-entertainment and social users do not exhibit significant effects.

Based on the above findings, the following policy recommendations are proposed to maximize the benefits of Internet use in delaying subjective aging among older adults.

First, Internet adoption among older adults should be expanded. The government should encourage businesses to develop age-friendly smart devices, increase the availability of Internet-enabled technologies, and enhance access to these devices among older individuals. Telecommunications companies should be guided to offer discounted broadband plans for seniors, lower the costs of home broadband and mobile data, and ease the financial barriers that hinder Internet access. Furthermore, the government should establish standardized digital skills training programs tailored for older adults, utilizing community centers, senior universities, and older adults care institutions to deliver training sessions. These initiatives would help close the access gap and contribute to lowering the subjective age of older adults.

Second, the effectiveness of Internet use among older adults should be enhanced. The government should optimize the provision of “Internet + public services” to improve access to social security, healthcare, and cultural engagement. ICT solution providers should be encouraged to develop health management applications, including health monitoring, telemedicine, and remote caregiving, to reduce healthcare costs for older adults. Additionally, society should foster a culture of filial piety by promoting “online family” platforms that facilitate intergenerational interaction and support. These efforts would strengthen the impact of Internet use on improving health status, self-efficacy, and social capital, thereby reducing subjective age among older adults.

Third, the utility gap among older adults should be bridged. The government should increase research and development subsidies to incentivize businesses to integrate cutting-edge technology into affordable commercial devices. Simultaneously, efforts should be made to improve digital infrastructure, accelerate the deployment of fiber-optic broadband and mid-to-high-frequency 5G networks, and enhance network transmission speeds. Additionally, content filtering technology should be utilized to identify and remove low-quality information, optimizing the digital experience for older users. These measures would help alleviate constraints to Internet use among rural and economically disadvantaged older individuals, narrowing the utility gap and lowering subjective age.

Fourth, the Internet use patterns among older adults should be optimized. The government should encourage Internet companies to promote diverse digital engagement among older users through non-coercive nudging strategies. Public awareness campaigns should also be strengthened to reshape perceptions of Internet use among older adults, encouraging participation in social, informational, and instrumental activities while reducing an overemphasis on entertainment-related usage. These efforts would help close the usage gap and further decrease subjective aging among older Internet users.

Finally, digital addiction among older adults should be prevented. While Internet use can help reduce older adults’ subjective age and enhance their quality of life, it may also lead to digital addiction, which can harm physical and mental health, increase feelings of loneliness, and ultimately diminish their overall well-being (54, 55). Therefore, alongside efforts to promote Internet use among older adults, it is essential to implement measures that prevent digital addiction in order to fully realize the benefits of the Internet. Existing studies have shown that digital addiction among older adults is influenced by several factors, including digital literacy, platform manipulation, and social support (56–58). In addition, the risk of digital addiction is likely to rise as the cost of Internet access continues to decrease (56). In response, the government should develop digital literacy training programs tailored to older adults, aiming to raise awareness of the potential harms of digital addiction and to encourage the formation of healthy Internet usage habits. Simultaneously, ICT solution providers should take into account the risks of digital addiction among older users by enhancing the age-friendliness of digital platforms and designing anti-addiction systems to help prevent irrational or excessive Internet use. Additionally, family members should increase their everyday interactions with older adults to reduce excessive reliance on the Internet. This can mitigate the risk of digital addiction among older adults, enabling a more balanced approach that maximizes the positive impacts of Internet use while minimizing its adverse effects, thereby improving the overall quality of life for older adults in the digital age.

## Data Availability

Publicly available datasets were analyzed in this study. This data can be found at: http://class.ruc.edu.cn/.
